# 113. Knowledge, Attitudes, and Practices towards HPV vaccination among reproductive-age women in an HIV hotspot in the US

**DOI:** 10.1093/ofid/ofac492.191

**Published:** 2022-12-15

**Authors:** Aasith Villavicencio Paz, Nicholas Fonseca Nogueira, Gray Kelsey, Julia Zukerberg, Ana S Salazar, Lucila Hernandez, Patricia Raccamarich, Deborah L Jones, Maria L L Alcaide

**Affiliations:** Hospital of the University of Pennsylvania, Philaldelphia, Pennsylvania; Division of Infectious Diseases, Department of Medicine, University of Miami Miller School of Medicine, Parkland, Florida; University of Miami Miller School of Medicine, Miami, Florida; University of Miami Miller School of Medicine,, Miami, Florida; Division of Infectious Diseases, Department of Medicine, University of Miami Miller School of Medicine, Parkland, Florida; Division of Infectious Diseases, Department of Medicine, University of Miami Miller School of Medicine, Parkland, Florida; Division of Infectious Diseases, Department of Medicine, University of Miami Miller School of Medicine, Parkland, Florida; University of Miami, Miami, Florida; Division of Infectious Diseases, Department of Medicine, University of Miami Miller School of Medicine, Parkland, Florida

## Abstract

**Background:**

Human papillomavirus (HPV) is the most common STI in the US, responsible for cervical cancer and increased risk of HIV acquisition. Despite an effective HPV vaccine, women’s HPV vaccination coverage and rates remain far below desired levels. This study aimed to evaluate HPV knowledge, screening, and vaccination practices as well as factors associated with HPV vaccination among women of reproductive age living in Miami, Florida, a Southern US city with a high incidence of STIs and low HPV vaccination coverage.

**Methods:**

In April 2022, HIV-negative, cisgender, sexually active women aged 18-45 years were referred from a study evaluating HIV risks. Surveys assessed socio-demographics and sexual behaviors. Validated questionnaires assessed HPV knowledge, screening, and vaccination practices. A cumulative HPV knowledge score was generated. Factors associated with HPV vaccination were analyzed by Chi-square, Fisher’s exact test, studentized t-test, and multivariate logistic regression (MLR).

**Results:**

A total of 67 participants were enrolled, and 54 who knew their vaccination status were included in the analysis: median age was 26 (IQR 22-34.5) years; 50% were White, 26% Black, and 33% were Hispanic. Median age at first sexual encounter was 18 (16-18) years, the mean number of sexual partners in the previous month was 1.45 (±1.44), and 33% had previous pregnancies. Mean HPV score was 13.4 (±8.84) out of 29, 43% reported a history of HPV/Pap smear screening. Barriers to HPV vaccination included: 28% low-risk perception, 24% healthcare barriers, and 41% vaccine hesitancy. In MLR, a one-point increase in HPV knowledge score increased the odds of vaccination by 21.5% (0.8-3.67%; p< 0.01). Age, number of sexual partners, and pregnancy history were not significant predictors of HPV vaccination.

**Conclusion:**

Findings suggest low HPV knowledge among women of reproductive age in a high-risk area, and suggest that increasing knowledge may reduce barriers to HPV vaccination and increase vaccine uptake.

**Disclosures:**

**Maria L L. Alcaide, MD**, Gilead: Advisor/Consultant.

